# Factor X-activating activity from Lewis lung carcinoma.

**DOI:** 10.1038/bjc.1980.109

**Published:** 1980-04

**Authors:** P. Hilgard, P. Whur


Br. J. Cancer (1980) 41, 642

### Short Communication

### FACTOR X-ACTIVATING ACTIVITY FROM LEWIS LUNG CARCINOMA

P. HILGARD* AND P. WHUR†

From the *Department of Haematology, Royal Postgraduate Medical School, London W12, and the †Cell Biology Unit, Marie Curie Memorial Foundation, Oxted, Surrey

Received 10 December 1979 Accepted 2 January 1980

THE PROCOAGULANT ACTIVITY elicited by some experimental tumours was recently characterized as a Factor X-activating enzyme (Curatolo *et al.*, 1979) and it was shown that this thromboplastic activity was clearly different from the Factor VII-dependent activity of most other tissues (Gordon *et al.*, 1975). Since this activity appeared in serum-free media of fibroblasts after chemical transformation, it was suggested that it might even represent a marker of malignancy (Gordon & Lewis, 1978).

Factor X-activating activity was determined in two established Lewis lung carcinoma (3LL) cell lines which were free of any contaminating host cells. Washed cells from cultured primary tumours and spontaneous lung metastases were transferred to serum-free media containing unsupplemented Dulbecco's medium without indicator and 20 mg/ml human Factor IX complex (prepared by the Plasma Fractionation Laboratory, The Churchill Hospital, Oxford). This preparation (Batch No. 9D1250) contained negligible amounts of Factor VII, 36 iu Factor IX, 52 u Factor II and 30 u Factor X/ reconstituted ml. The chromogenic substrate S-2222 (Kabi Vitrum Ltd, London) was added to the culture medium in a final concentration of 1 mM. After 2 h of incubation the optical density was read at 405 nm in a spectrophotometer. Appropriate controls consisted of cell-free as well as Factor X-free samples. The positive control contained 50 mg/ml Russell's Viper venom instead of tumour cells. The results were expressed as arbitrary units derived from the optical densities generated by $2{\cdot}7 \times 10^5$ cells; they are summarized in Table I. As can be seen, only Russell's Viper venom, which is known to be a potent direct activator of Factor X, and tumour cells, produced appreciable amounts of proteolytic activity. There was no statistical difference between the levels of Factor X activator detected in cultured primary and metastatic cells.

After transplantation of freshly suspended 3LL cells derived from a primary tumour into the upper thigh of 12 C57BL mice, 6 animals were treated with the vitamin K antagonist phenprocoumon throughout tumour growth, as previously described (Hilgard *et al.*, 1977). Twelve days after transplantation the primary tumour was surgically removed and the animals were allowed to survive until Day 29. They were then killed and the secondary lung tumours carefully excised. The primary tumours, lung tumours and fragments of abdominal muscle were homogenized and sonicated in normal saline. The protein content of each tissue extract was adjusted to 20 g/l with saline. all extracts were tested for clot-promoting activity using Factor VII-, Factor VIII- and Factor X-deficient human plasma as described by Curatolo *et al.* (1979).

Table II gives the mean clotting times obtained with different tissue extracts. Considerable thromboplastic activity of all tissues was found when tested in

TABLE I.—*Factor X-activating activity measured with chromogenic substrate in arbitrary units derived from optical densities (OD)*

| Test sample* | Factor X | OD units | ±s.e. |
|---|---|---|---|
| Culture medium | + | 0·4 | ±0·04 |
| Lung cells | − | 0·35 | ±0·00 |
| Primary cells | − | 0·14 | ±0·01 |
| Russell's Viper venom | + | 2·00 | — |
| Lung cells | + | 1·24 | ±0·02 |
| Primary cells | + | 1·09 | ±0·06 |

* All test samples contained equal volumes of culture medium.

TABLE II.—*Clot-promoting activity of various tissue extracts tested in human plasma deficient in specific clotting factors*

| | Clotting times (range in sec) Plasma deficient in | | |
|---|---|---|---|
| | Factor VII | Factor VIII | Factor X |
| Muscle (control) | 73–92 | 18–24 | 182–196 |
| Muscle (phen)† | 79–89 | 19–26 | 178–201 |
| Primary tumour (control) | 24–32 | 20–29 | 161–185 |
| Primary tumour (phen)† | 39–54* | 25–38 | 171–212 |
| Metastases (control) | 26–35 | 22–36 | 190–213 |
| Metastases (phen)† | 44–63* | 24–40 | 186–209 |
| Saline | 85–104 | 105–128 | 212–226 |

* Significantly different ($P < 0{\cdot}01$) from corresponding control.

† Phen = tissue from phenprocoumon-treated animals.

Factor VIII-deficient plasma. In contrast, none of the extracts showed activity when added to Factor X-deficient plasma. Distinct differences however were found when these tissue extracts were tested in Factor VII-deficient plasma; muscle extracts were almost inactive, whereas extracts from primary tumour and lung metastases of untreated animals produced very short clotting times. Extracts from primary and secondary tumours derived from phenprocoumon-treated animals, however, showed considerably less enzyme activity.

From the foregoing it is evident that single-cell cultures from primary and secondary 3LL show a Factor X-activating activity when tested by chromogenic substrates. A similar activity can be found in aqueous extracts of solid tumours and metastases using test plasma with specific clotting factor deficiencies. It is interesting to note that phenprocoumon treatment of the tumour-bearing animals produced a significant decrease of Factor X-activating activity, indicating that this proteolytic enzyme is vitamin-K-dependent. No significant differences between primary and secondary tumours could be established by either method.

The biological significance of the described procoagulant activity in cancer cells is unknown. Previous investigations have shown that warfarin (Poggi *et al.*, 1978), phenprocoumon (Hilgard *et al.*, 1977) and diet-induced vitamin K deficiency (Hilgard, 1977) inhibit primary tumour growth as well as the formation of spontaneous metastases of 3LL. The decreased synthesis of the procoagulant, Factor X-activating activity in anticoagulated and vitamin K-deficient animals indicates that the presence of this enzyme in tumour cells might facilitate the primary and metastatic growth of malignant tumours. This assumption accords with the current concept of the role of fibrin formation in tumour growth (Roos & Dingemans, 1979) and provides an additional rationale for the use of vitamin K antagonists in cancer therapy.

### REFERENCES

CURATOLO, L., COLUCCI, M., CAMBINI, A. L. & 4 others (1979) Evidence that cells from experimental tumours can activate coagulation Factor X. *Br. J. Cancer*, **40**, 228.

GORDON, S. G., FRANKS, J. J. & LEWIS, B. (1975) Cancer Procoagulant A: A Factor X activating procoagulant from malignant tissue. *Thromb. Res.*, **6**, 127.

GORDON, S. G. & LEWIS, B. J. (1978) Comparison of procoagulant activity in tissue culture medium from normal and transformed fibroblasts. *Cancer Res.*, **38**, 2467.

HILGARD, P. (1977) Experimental vitamin K deficiency and spontaneous metastases. *Br. J. Cancer*, **35**, 891.

HILGARD, P., SCHULTE, H., WETZIG, G., SCHMITT, G. & SCHMIDT, C. G. (1977) Oral anticoagulation in the treatment of a spontaneously metastasizing murine tumour. *Br. J. Cancer*, **35**, 78.

POGGI, A., MUSSONI, L., KORNBLIHTT, L., BALLABIO, E., DE GAETANO, G. & DONATI, M. B. (1978) Warfarin enantiomers, anticoagulation and experimental tumour metastasis. *Lancet*, i, 163.

ROOS, E. & DINGEMANS, K. P. (1979) Mechanisms of metastasis. *Biochim. Biophys. Acta*, **560**, 135.


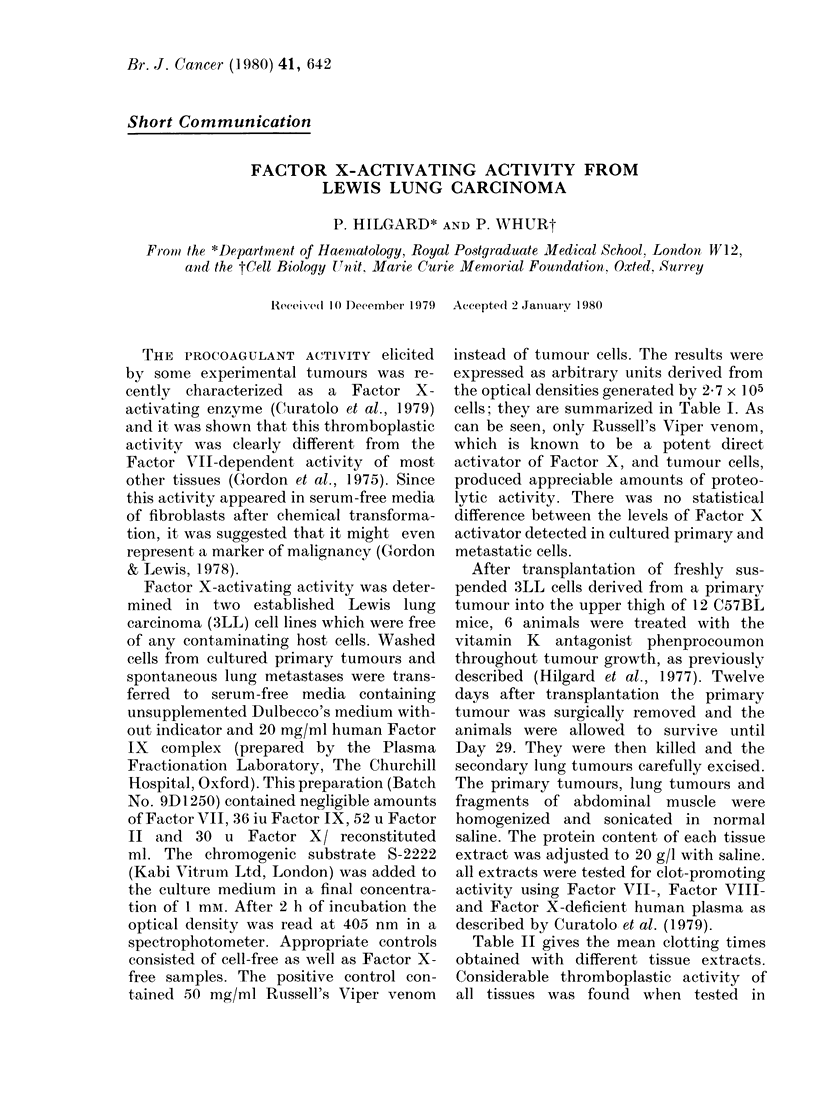

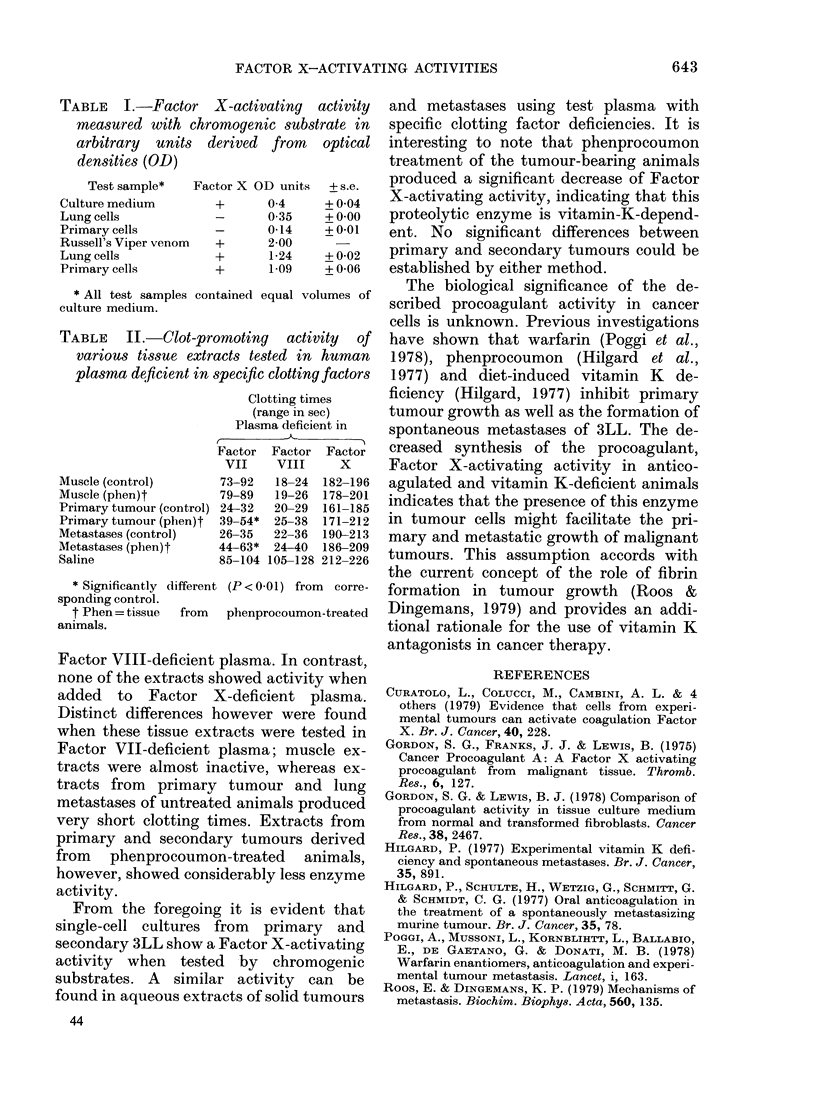


## References

[OCR_00205] Curatolo L., Colucci M., Cambini A. L., Poggi A., Morasca L., Donati M. B., Semeraro N. (1979). Evidence that cells from experimental tumours can activate coagulation factor X.. Br J Cancer.

[OCR_00211] Gordon S. G., Franks J. J., Lewis B. (1975). Cancer procoagulant A: a factor X activating procoagulant from malignant tissue.. Thromb Res.

[OCR_00217] Gordon S. G., Lewis B. J. (1978). Comparison of procoagulant activity in tissue culture medium from normal and transformed fibroblasts.. Cancer Res.

[OCR_00223] Hilgard P. (1977). Experimental vitamin K deficiency and spontaneous metastases.. Br J Cancer.

[OCR_00228] Hilgard P., Schulte H., Wetzig G., Schmitt G., Schmidt C. G. (1977). Oral anticoagulation in the treatment of a spontaneously metastasising murine tumour (3LL).. Br J Cancer.

[OCR_00234] Poggi A., Mussoni L., Kornblihtt L., Ballabio E., de Gaetano G., Donati M. B. (1978). Warfarin enantiomers, anticoagulation, and experimental tumour metastasis.. Lancet.

[OCR_00240] Roos E., Dingemans K. P. (1979). Mechanisms of metastasis.. Biochim Biophys Acta.

